# Incidence, complications and therapeutic evaluation of clinical hypothyroidism in different breeds of dogs

**DOI:** 10.1186/s12917-025-04746-4

**Published:** 2025-05-09

**Authors:** Gehad E. Elgalfy, Mohamed M. Ghanem, Mahmoud A. Y. Helal, Heba M. El-khaiat

**Affiliations:** Animal Medicine Department, Faculty of Veterinary Medicine, Benha University, Po Box: 13736, Moshtohor, Toukh, Egypt

**Keywords:** Complications, Dermatological changes, Dogs, Hypothyroidism, Levothyroxine, Obesity

## Abstract

**Background:**

Hypothyroidism is one of the most common endocrine disorders in dogs, that is caused by reduction in thyroxine hormone production.

**Objectives:**

This study aimed to detect the incidence of clinical hypothyroidism among cases suffering from dermatological changes or obesity, investigate the complications in severely affected hypothyroid dogs and evaluate the response to levothyroxine treatment.

**Method:**

Total number of 212 dogs of different ages, breeds and of both sexes were included in this study, where 200 dogs were suffering from alopecia or obesity, the other 12 healthy dogs were used as control group.

**Results:**

After ruling out other causes of alopecia and obesity, hypothyroidism was diagnosed in 28 dogs (14%) depending on the result of thyroid function test. Results of this study showed that the highest incidence of hypothyroidism was reported in Golden retriever (21.42%) followed by Griffon dogs (17.85%). Additionally, hypothyroidism was mostly reported in middle-aged dogs (60.71%) of different breeds. Males (57.14%) revealed higher incidence than females. The hypothyroid dogs showed a wide range of clinical signs including dermatological, metabolic, psychological alterations in addition to cardiovascular, respiratory, neuromuscular, gastrointestinal and gynecological abnormalities. Radiography revealed pulmonary edema in dogs affected with heart problems (*N* = 2), dilated esophagus in hypothyroid dogs showing vomiting (*N* = 2). Echocardiographic examination of hypothyroid dogs with heart problems revealed first grade diastolic dysfunction. Treatment with levothyroxine induced clinical improvement within one month of administration.

**Conclusion:**

Hypothyroidism is a relatively common condition in dogs compared to other diseases causing dermatological changes or obesity in dogs. Therefore, it must be considered for early diagnosis and treatment before encountering complications that could be life threatening for dogs.

## Background

Clinical hypothyroidism is the most common endocrine disorder in dogs coming second to diabetes mellitus, characterized by reduction in thyroxine hormone production as a result of either thyroidal cause (primary) or non-thyroidal cause (secondary or tertiary) due to defect in pituitary gland or hypothalamus respectively [1, 2].

Primary hypothyroidism is the most common cause of naturally occurring thyroid dysfunction in dog, reported in more than 95% of affected cases. Two histologic forms of primary hypothyroidism are recognized in dogs- lymphocytic thyroiditis and idiopathic atrophy. Other much rarer causes include iodine deficiency, goitrogen ingestion, congenital hypothyroidism, thyroid gland destruction by neoplasia, drug therapy, surgical thyroidectomy, and treatment with radioactive iodine [3].

The progression of thyroid disease is often slow, and clinical signs may not be noticeable until a significant portion of the thyroid gland is affected, typically around 75%. As thyroid hormones play a crucial role in regulating metabolism and influencing numerous organ systems, clinical signs of hormone deficiency are broad and unspecific, including metabolic disorders, dermatological alterations which are the most encountered in clinics. Neurological and other less common signs such as hypothermia, cardiac defect, ocular changes and GIT disorders are also reported [4].

The major dermatological alterations include non- pruritic, non- inflammatory, bilaterally symmetrical alopecia that mostly appears on the dorsum of the tail (rat tail appearance), dry or oily seborrhea, hyperpigmentation, thickened skin (myxedema) and skin comedones [5].

Diagnosis of hypothyroidism depends on the observation of any of the specific clinical signs of hypothyroidism, followed by measurement of thyroid hormone level that represented by increased serum concentrations of thyroid stimulating hormone (TSH) and decreased T4 concentration [6, 7].

Levothyroxine (LT4) has long been regarded as the standard treatment for hypothyroidism. When taken orally, this medication is effective, has a prolonged serum half-life that allows for once-daily dosing, and typically leads to the resolution of hypothyroidism symptoms in most patients [8].

Therefore, this study was designed to screen the occurrence of clinical hypothyroidism in different breeds of dogs, suffering from dermatological changes or being obesity. Moreover, we track the cases for detecting complications in severely affected hypothyroid dogs and evaluate the response to levothyroxine therapy.

## Methods

### Animals

This survey study was carried out during the period from August 2022 to August 2024. It was applied on a total number of 212 clinical cases of dogs of different breeds, ages and of both sexes. That clinical cases were admitted to pet animal veterinary clinic of Faculty of Veterinary Medicine, Benha University, private pet animal clinics and different pet animal’s shelters located in Cairo and Giza governorate, Egypt. The selection criteria for the 200 dogs included in this study were as follows: adult dogs over 2 years of age, showing signs suggestive of hypothyroidism, such as dermatological changes or obvious obesity. On the other hand, twelve apparently healthy dogs were used as control based on the result of clinical, hematological (CBC) examinations, liver, kidney and heart function test and lipid and protein profiles as well as thyroid function test.

The affected dogs with alopecia (*N* = 193) were subjected to clinical and physical examination, skin scraping, UV lamb, fecal examination and bacteriological examination to rule out other causes of alopecia rather than endocrinal disorders. The exclusion criteria were including the dogs having non-thyroidal illnesses or were receiving any drugs that could affect thyroid function within the 6 months prior to the study. After ruling out other causes, 29 cases with alopecia, in addition to 7 cases with obesity were subjected to initial screening test for hypothyroidism by measuring TT4. Only 28 dogs showed a reduced TT4 level. Further confirmation was required by evaluation of FT4, TSH and TgAA levels. Based on clinical signs and the results of diagnostic tests, the diagnosis of hypothyroidism is confirmed in 28 dogs.

The severely affected hypothyroid dogs were subjected to further investigations by radiography, ultrasonography and echocardiography to detect other thoracic, abdominal and cardiac changes that could be associated with hypothyroidism in dogs.

### Samples

Blood samples were collected from each dog from the cephalic vein, without anticoagulant, clotted at room temperature for 20 min, centrifuged at 3,000 rpm for 10 min, and then the clear non-hemolyzed serum samples were separated and stored at -20°c for further analysis.

### Thyroid function test

Serum samples were used to determine TT4 levels using veterinary specific kits (Catalyst total T4 slide) and Catalyst One Chemistry Analyzer, IDEXX laboratories, Inc, Westbrook, Maine, according to the manufacturer’s instruction, for screening incidence of hypothyroidism in the examined dogs. FT4 and TSH were measured by Enzyme linked fluorescent assay, [VIDAS, Biomerieux] [9, 10]. Thyroglobulin autoantibodies (TgAA) were evaluated by enzyme-linked immunosorbent assay techniques as previously [11].

### Prolactin and estrogen hormone assessment

The prolactin and estrogen hormone levels in canine serum samples were evaluated using the commercial canine ELISA kits (MyBioSource, USA and E-OSEL-C0001, USA) according to the manufacturer’s instructions.

### Radiographic examination

Radiographic evaluation of the lung fields is enhanced by exposing the radiograph at maximum inspiration if possible. As previously described [12].

### Echocardiographic examination

Echocardiographic examination for severely affected hypothyroid dogs was performed by using Mindray M9 ultrasound machine (made in China) with P10-4s probe, as previously described [13].

### Treatment protocol

The hypothyroid dogs were treated with Levothyroxine tab. (Euthyrox 100 µg^®^ or Eltroxin 100 µg^®^), as an initial dose 0.01 mg/kg twice daily. Dogs were revaluated one month after initiating therapy and the dosage was adjusted based on the clinical response, and result of serum TT4 analysis. Treatment of hypothyroidism consists of lifelong administration of synthetic levothyroxine after the dose was adjusted [8, 14].

### Statistical analysis

The statistical analysis was carried out using One-way ANOVA using SPSS, ver. 25 **(IBM Corp. Released 2013)**. Data were treated as a complete randomization design according [15]. Multiple comparisons were carried out applying Duncun test. The significance level was set at *P* < 0.05.

## Results

### Clinical examination

Data was collected from 200 dogs of adult ages with history of alopecia or obesity. After ruling out other causes of alopecia (mange, parasitic infestation, ring worm, skin allergy, pyoderma and environmental cause) and the causes of obesity (Cushing’s syndrome and overfeed), only 28 dogs were diagnosed as hypothyroid depending on presence of clinical signs of hypothyroidism and the confirmatory result of thyroid function tests. The incidence of canine hypothyroidism in the included cases was 14%.

Data in (Table 1**)** showing that the incidence of hypothyroidism among the affected dogs included in the study was 8.37%, 78.57% and 28.57% of the affected cases with signs of alopecia, alopecia with obesity and obesity, respectively.

**Table 1:** The incidence of hypothyroidism among cases of alopecia and obesity included in this study

Signs	Number (*N* = 200)	causes	Number	Percent %
1-Alopecia	179	hypothyroidism	15	8.37%
		Mange	42	23.46%
		Ring worm	34	18.99%
		Parasitic infestation	62	34.63%
		Skin allergy	30	16.75%
		Pyoderma	5	2.79%
		Environmental cause	9	5.02%
2-Alopecia with obesity	14	hypothyroidism	11	78.57%
		Cushing’s syndrome	3	21.42%
3-Obesity	7	hypothyroidism	2	28.57%
		over feeding	5	71.42%

Incidence of hypothyroid cases on the basis of age, breed and gender of dogs is summarized in (Table 2). In this study hypothyroidism was mostly reported in middle aged (*N* = 17 dogs, 60.71%) followed by young aged (*N* = 6 dogs, 21.42%) and old aged (*N* = 5 dogs, 17.85%) dogs of different breeds. The highest incidence was in Golden retriever (*N* = 6 dogs, 21.42%) followed by Griffon (*N* = 5, 17.85%), German shepherd and Labrador retriever (*N* = 4, each 14.28%) Rottweiler, Balady dogs and Pitbull (*N* = 2, each 7.14%) and Beagale, Pomeranian and Bulldog (*N* = 1 each, 3.57%). Males (*N* = 16, 57.14%) revealed higher incidence to hypothyroidism than females (*N* = 12, 42.85%).

**Table 2:** Incidence of hypothyroidism based on age, breed and gender of dogs

	Age	
Age (years)	Number of dogs	Percent
Young aged (> 5years)	6	21.42%
Middle aged (5–10 years)	17	60.71%
old aged (< 10 years)	5	17.85%
Breeds		
Breed	Number of dogs	Percent
Golden retriever	6	21.42%
Griffon	5	17.85%
German shepherd	4	14.28%
Labrador retriever	4	14.28%
Pitbull	2	7.14%
Rottweiler	2	7.14%
Balady dogs	2	7.14%
Pomeranian	1	3.57%
Begal,	1	3.57%
Bulldog	1	3.57%
Gender		
Gender	Number of dogs	Percent
Males	16	57.14%
Females	12	42.85%

**Fig. 1:**
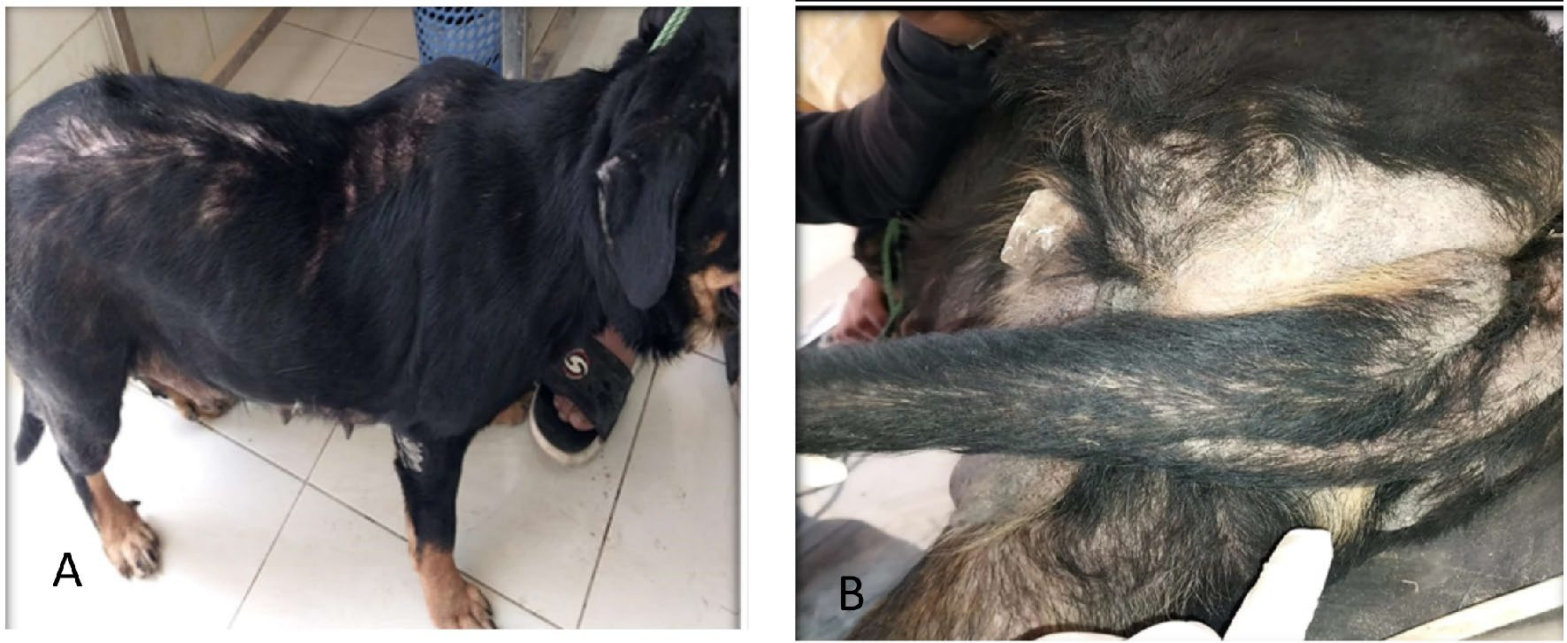
Bilateral symmetrical alopecia (**A**) and alopecia over area of excessive wear (base of tail and tail) giving rat tail appearance (**B**) in Rottweiler dog affected with hypothyroidism

Incidence of different clinical features in the hypothyroid dogs is summarized in (Table 3). The hypothyroid dogs showed variable clinical signs such as metabolic changes (weight gain despite normal appetite), psychological alterations (depression and lethargy). and dermatological changes (non-inflammatory non-pruritic alopecia). Asymmetric alopecia over area of excessive wear such as caudal thigh, base of tail and tail giving rat-tail appearance. These changes were reported in mild cases. While in severe cases, bilateral symmetrical truncal alopecia was reported (Fig. 1). Patchy alopecia was reported in one dog. Three cases showed skin pigmentation and skin comedones. Most cases showed normal pulse, respiratory rate and body temperature.

**Table 3:** Incidence of different clinical findings of hypothyroidism in the affected dogs

Clinical signs	Hypothyroid cases (*N* = 28)	Percent(%)
Dermatological changes (alopecia)	26	92.85%
Metabolic alterations (obesity)	13	46.42%
Psychological alterations (depression and lethargy)	12	42.85%
Gynecological defects (infertility)	4	14.28%
Signs related to CNS (ataxia and hind limb paresis)	3	10.71%
Signs related to GIT(vomiting)	2	7.14%
Signs related to CVS(edema, cough, exercise intolerance)	2	7.14%

Problems that were reported in severely affected hypothyroid dogs in this study include skin abnormalities such as [myxedema (*N* = 2), pyoderma (*N* = 2), generalized alopecia (*N* = 2)], cardiovascular and respiratory abnormalities (edema, exercise intolerance, cough, labored respiration) (*N* = 2), neuromuscular (ataxia and hind limb paresis) (*N* = 3), gastrointestinal (vomiting) (*N* = 2) and infertility problems (*N* = 4).

**Fig. 2:**
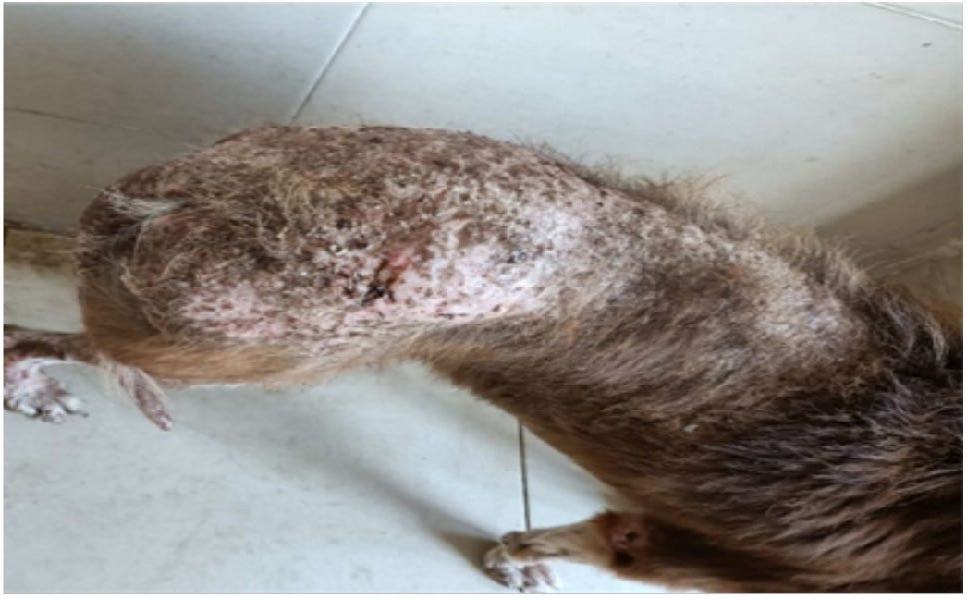
Bilateral symmetrical alopecia with pyoderma, seborrhea and dry brittle hair in dog affected with hypothyroidism

**Fig. 3:**
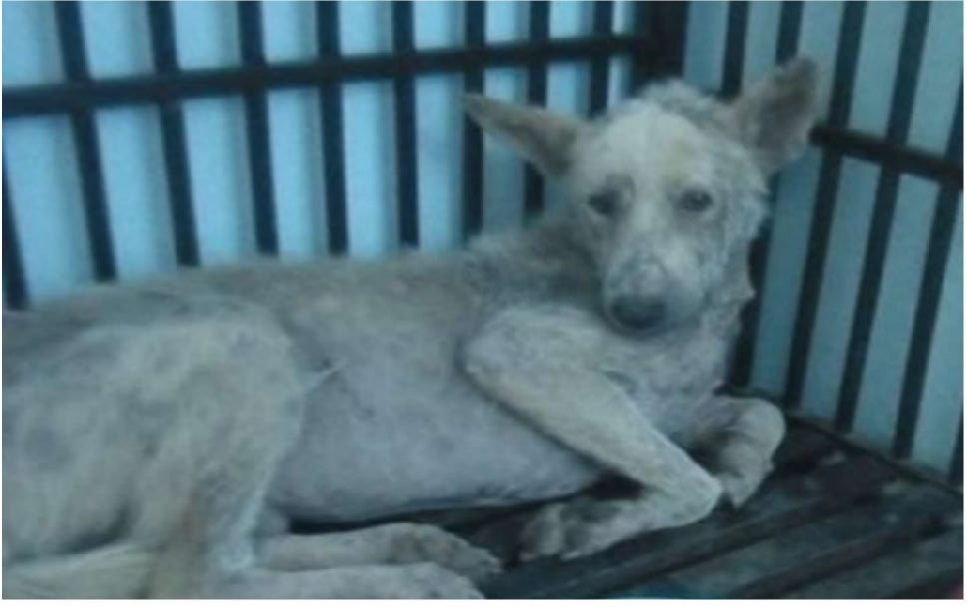
Generalized alopecia in dog affected with hypothyroidism

Myxedema (thickened skin) was reported in two cases in different parts mainly throat area and face that giving the classical tragic face appearance of hypothyroidism. Two cases suffered from pyoderma and seborrhea in different areas of skin such as hindquarter, trunk, head and limbs, that was admitted to the clinic with history of severe recurrent skin lesion that affect the hind quarter and trunk, with dry and brittle hair which not respond to any classical treatment (Fig. 2). Another two cases were suffered from generalized alopecia (Fig. 3).

Hypothyroid dogs affected with cardiovascular and respiratory diseases (*N* = 2) showed signs of depression, lethargy, exercise intolerance, obesity, patchy alopecia and rat tail appearance, in addition to edema around the neck and face, easily tired after a simple activity, cough, cyanosis, deep labored respiration, and crackles during chest examination.

Neuromuscular signs were reported in three cases in form of ataxia and hind limb paresis. The digestive disorder included weight loss and vomiting immediately after having a meal or water, suggesting for megaesophagus.

Infertility problems were reported in four females with different forms of infertility such as anestrum for two years or lack of conception for three years and galactorrhea.

### Laboratory findings

#### Thyroid function test

The hypothyroid dogs showed a significant decrease in TT4 and FT4 levels and a significant increase in TSH level compared to control dogs (Table 4). TgAA were positively detected in seven hypothyroid dogs.

#### Skin microbiological examination

From the microbiological examination of a dermal sample of hypothyroid dogs with pyoderma, Proteus spp. and Methicillin sensitive coagulase negative Staphylococcus were isolated.

#### Prolactin and Estrogen levels in infertile hypothyroid dogs

The infertile hypothyroid cases showed a significant increase (*P* > 0.05) in prolactin level (11.01 ± 0.11 Ng/mL) [control 7.27 ± 0.49] and a significant decrease (*P* > 0.05) in estrogen level (0.05 ± 0 pg/mL) [control 0.2 ± 0.06] compared to control dogs.

#### Radiographic examination

**Fig. 4:**
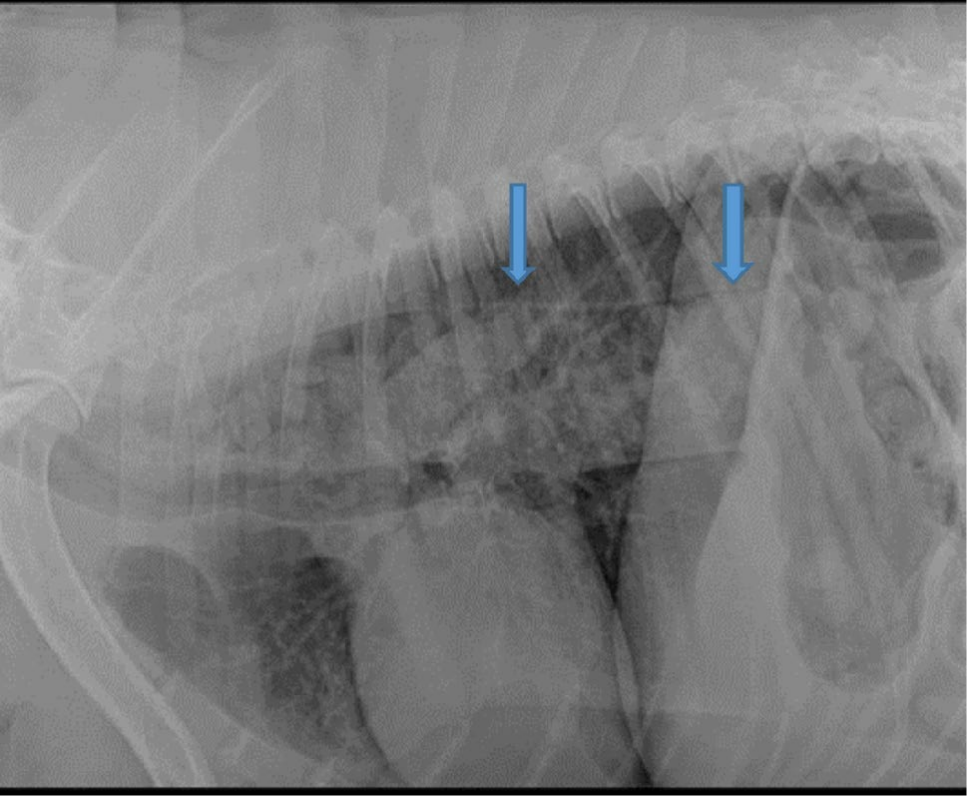
Thoracic radiograph shows dilated esophagus in hypothyroid dog

Chest radiographic examination of severely affected hypothyroid dogs revealed a diffuse increased alveolar and interstitial pattern that was consistent with pulmonary edema. These hypothyroid dogs were suffering from signs of respiratory and heart problems. In addition, radiography of vomiting dogs illustrated dilation of esophagus (megaesophagous) Fig. 4.

#### Echocardiographic examination

**Fig. 5:**
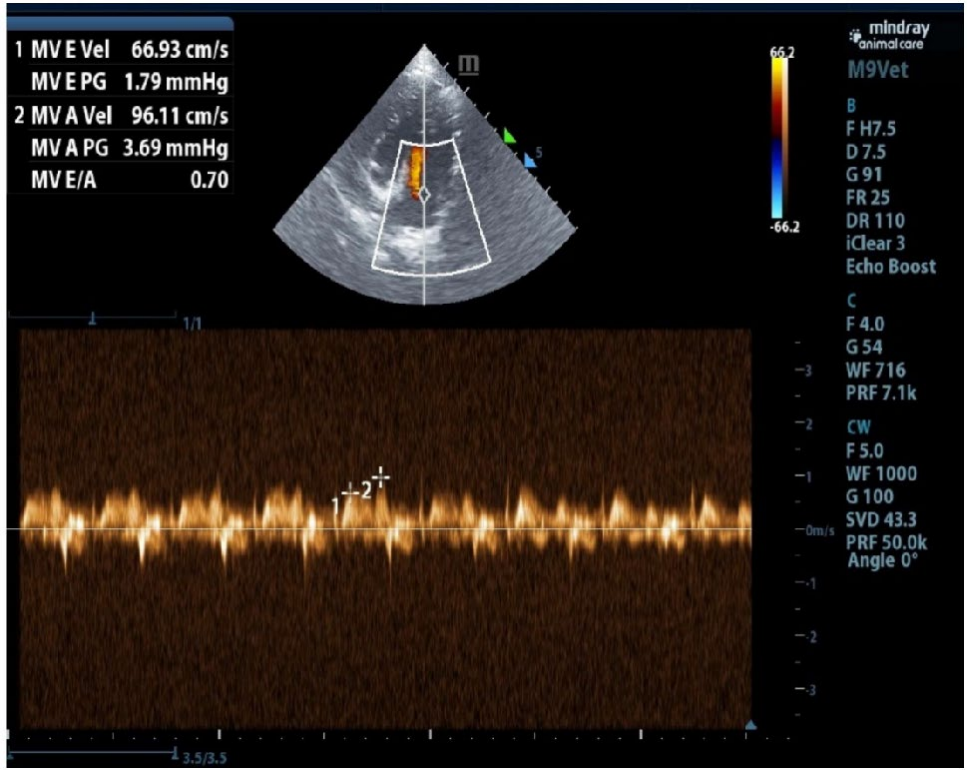
Echocardiographic view shows a reversed E/A ratio, equal to 0.7 indicate first grade diastolic cardiac dysfunction in hypothyroid dog

Echocardiographic examination of severely affected hypothyroid dogs revealed a reversed E/A ratio [ E/A ratio = 0.7] that suggest first grade diastolic cardiac dysfunction (Fig. 5).

#### Evaluation of levothyroxine treatment

After one month of levothyroxine administration, TT4 and FT4 levels were increased while TSH level decreased compared to pre-treatment levels (Table 4**)**. Mental and physical activity were improved within the first week of treatment, while dermatological changes and body weight were improved within several months. Hair growth began within the first month, with complete regrowth after about 6 to 8 months. The body weight changes began within 5 to 8 weeks and completely improved after 6 months of treatment.

**Table 4:** Thyroid function test in control, hypothyroid dogs before and after treatment. (mean ± SE)

Parameter	Control *N* = 12	Hypothyroid *N* = 28	After treatment *N* = 21	*P*-value	Threshold Value
TT4	1.92 ± 0.13^**A**^	0.63 ± 0.04^**B**^	1.67 ± 0.08^**A**^	0.000	0.8–3.6 µg/dl
Ft4	1.63 ± 0.10^**A**^	0.93 ± 0.29^**B**^	1.43 ± 0.19^**AB**^	0.056	1.0–2.5 ng/dl
TSH	11.38 ± 0.52^B^	34.05 ± 2.27^**A**^	9.33 ± 1.58^**B**^	0.000	0–37 mlu/L

A & B: There is no significant difference (*P* > 0.05) between any two means, within the same row have the same superscript letter

TT4: Total tetraiodothyroxin, FT4: Free thyroxine, TSH: Thyroid stimulating hormone

## Discussion

Hypothyroidism has been considered one of the most common endocrine disorders in dogs during the past few years, difficult in diagnosis but very easy to be treated. Definitive diagnosis of hypothyroidism in dogs requires presence of the characteristic features of the disease and using of thyroid function diagnostic tests which may be difficult to interpret due to presence of non-thyroidal illness or recent drug administration, so any animal having one of these characters was excluded from the study [3].

This study aimed to detect the incidence of clinical hypothyroidism among cases suffering from dermatological changes or obesity which are considered the most common clinical signs of hypothyroidism in dogs.

Alopecia and hair loss in dogs can indeed be a major concern for both pet owners and veterinarians. These conditions can range from mild, temporary issues to more serious, chronic health problems. The causes behind alopecia in dogs are varied, and determining the main cause can sometimes be challenging. The dermatological examination should include a careful examination of the skin and the coat conditions [16].

Usually, alopecia caused by endocrinal disorders is initially non-pruritic but may become pruritic because of secondary, concurrent bacterial or ectoparasite infestations. The presence of thickened (myxedema) and hyperpigmented skin and comedones without inflammation is usually suggestive of hypothyroidism [16].

Causes of alopecia rather than endocrinal disorders (hypothyroidism and Cushing’s syndrome) that were reported in this study include mange, parasitic infestation, ring worm, skin allergy, pyoderma and environmental causes, similar results were previously reported [17]. While hypothyroidism and Cushing’s syndrome were reported in (13.47%) and (1.55%) of affected dogs that were included in this study respectively.

TT4 measurement is used as screening test for hypothyroidism, it has higher sensitivity more than 90% and specificity of 75–80% [18]. Further confirmation was required by evaluation of FT4 and TSH levels. The hypothyroid cases in our study showed a significant decrease in TT4 and Ft4 levels and a significant increase in TSH level, the same results were previously published [19–21].

Measuring free thyroxine (FT4) detects the concentration of free hormone available to tissue that showing high correlation with the clinical state of the animal. It is less sensitive than TT4 for diagnosis of hypothyroidism as about 20% of dogs have value with low normal reference interval, but more specific as it is less affected by NTI [18].

Increased TSH level referred to a lack of negative feedback on the pituitary gland from thyroid hormones. Falsely excluding hypothyroidism due to a TSH result within the reference interval is one of the most common errors in diagnosing hypothyroidism. Although the combination of a low TT4 and a high TSH is relatively specific for diagnosing hypothyroidism, TSH can be within the normal reference interval in 15 to 40% of hypothyroid cases [21, 22].

T3 (triiodothyronine) has a greater biological activity than T4 and plays a more significant role in regulating metabolism. However, the use of total T3 measurements has low sensitivity and accuracy to diagnose canine hypothyroidism as a significant portion of circulating T3 is derived from the peripheral conversion of T4 into T3 in different tissue rather than being directly produced by the thyroid gland [6].

The most common cause of hypothyroidism is lymphocytic thyroiditis that is an autoimmune inflammatory disorder caused by the presence of thyroglobulin auto antibodies. In this study, TgAA were positively detected in seven hypothyroid dogs. Its measurement was used to investigate within reference interval TT4 concentrations in dog strongly suspected to be hypothyroid as interference from TgAA give false normal TT4 [18]. In addition, TgAA measurements are crucial for identifying autoimmune thyroiditis, a common underlying cause of primary hypothyroidism in dogs [7]. Based on clinical signs and the results of diagnostic tests, the diagnosis of hypothyroidism is confirmed in affected dogs, as previously reported [7, 9, 18].

In our study, the highest incidence of hypothyroidism was reported in Golden retriever followed by Griffon, German shepherd and Labrador retriever dogs. similar observations were previously described by Ryad et al. [9] who reported that maximum number of cases were observed in Golden retriever, followed by German Shepherd and Rottweiler dogs and Milne and Hayes Jr [23]. who stated that nine breeds of dogs were found to be at high-risk for hypothyroidism, mentioned firstly Golden retrievers.

In this study, hypothyroidism was mostly reported in middle aged dogs of different breeds, as previously reported [10, 23, 24]. Males revealed higher incidence to hypothyroidism than females [10, 25].

Thyroid gland is responsible for the production of thyroid hormones which are necessary for function of most body systems, so the hypothyroid dogs reveal a wide range of clinical signs. In our study, dermatological and metabolic alterations were the most common clinical signs reported in the hypothyroid dogs. These changes were reported in 92.85% and 46.42% of the affected cases respectively, similar results were previously published [4, 26].

Thyroid hormones play an important role in the regulation of metabolic pathway through enhancing and diminishing energy consumption from different dietary resources like carbohydrate and lipid [27]. Its deficiency resulted in signs of decreased metabolic rate, represented by obesity despite normal or low appetite and exercise intolerance, that were reported in 46.42% of hypothyroid dogs in the present study. While the psychological alterations (lethargy and depression) were reported in 42.85% of the affected hypothyroid dogs [5, 19, 24].

In our study, dermatological changes caused by hypothyroidism were reported in 92.85% of the affected cases. These changes were represented by non-inflammatory non pruritic alopecia appeared in asymmetrical form in mild cases and distributed in different parts such as thigh, tail base and tail of the animal, while in severe cases appeared in form of symmetrical bilateral truncal alopecia. Some cases showed skin hyperpigmentation or skin comedones [24, 28, 29], this can be explained by the thyroxine hormone function in initiation and maintaining the anagen or growing phase of the hair cycle. Thyroxin hormone reduction results in premature entrance of hair follicles to the telogen phase, excessive shedding and lack of hair regrowth, resulting in alopecia [28].

Localized alopecia with a dry, brittle coat was reported mainly in mild cases of hypothyroidism when these cases become severely affected that induces hair loss and generalized alopecia was developed [30]. Thyroid hormones are responsible for the production of “proteoglycans” in the skin, which are linked to epidermal differentiation by activating fibroblasts and keratinocytes. So, generalized alopecia may develop in severe cases of the disease [30, 31].

In this study, pyoderma was reported in two of the hypothyroid cases that were admitted to the clinic with history of sever recurrent skin lesion, affecting the hind quarter and trunk, with dry brittle hair which not respond to any classical treatment [10, 19]. That attributed to addressing the dermal lesion directly without considering an underling cause, leading to treatment failure, lesion progression and bacterial resistance against antibiotics after repeated administrations [26]. Hypothyroidism was suspected when the cases not improved by any treatment, so thyroid function test was applied and showed decreased TT4, FT4 and increased TSH levels.

When the hypothyroid cases advance, other signs appear including skin abnormalities such as generalized alopecia, myxedema, pyoderma and other signs [32, 33]. Thus, this study aimed to give overview of the complications reported in severely affected hypothyroid dogs.

Hypothyroidism often is suspected and investigated as an underlying cause of skin problems only after various unsuccessful treatment protocols have been applied [20, 29]. From the microbiological examination of a dermal sample of hypothyroid dogs with pyoderma, Proteus spp. and Methicillin sensitive coagulase negative Staphylococcus were isolated. The thyroid gland plays a greater role in regulating several functions and influences the immune system and hence, when it is depressed, the whole body becomes more and more susceptible to the infections. This could be attributed to recurrent bacterial infections of the skin reported in the present study [29].

Other uncommon problems were reported in some of the severely affected hypothyroid dogs such as cardiovascular and respiratory disorder, neuromuscular, infertility and gastrointestinal disorders [4, 10].

Thyroid hormones modulate physiological functions of the cardiovascular system in various ways, affecting myocardial contractility, heart rate, and systemic vascular resistance, and thus both thyroid hormone excess and deficiency can be associated with a higher cardiovascular risk [34].

In this study, the hypothyroid dogs affected with heart problems showed signs of depression, lethargy, obesity, exercise intolerance, cough, cyanosis, deep labored respiration, and crackles during chest auscultation. Radiographic examination revealed pulmonary edema in two cases, as previously described [35].

The most common heart problem reported in hypothyroidism in dogs is dilated cardiomyopathy(DCM) [36, 37], but in this study the hypothyroid dogs with heart problem suffered from diastolic cardiac dysfunction that detected by echocardiographic examination which revealed a reversed E/A ratio equal to 0.70, suggest first grade diastolic cardiac dysfunction.

The E/A ratio is a ratio of early to late ventricular filling velocities, and a reduced E/A ratio signifies diastolic dysfunction from impaired relaxation. Consequently, it results in a state of low cardiac output with decreased heart rate and stroke volume [38].

Diastolic dysfunction refers to impaired relaxation and abnormal heart filling during diastole of left ventricle because it has stiffened overtime. The affected hypothyroid dogs suffered from pulmonary edema, that may have attributed to stiffness of left ventricle [39].

Hypothyroidism is associated with several neurological signs mentioned by many reports resulting from involvement of the peripheral nervous system, such as vestibular dysfunction, facial paralysis, reduced spinal reflexes, ataxia, paresis and moving in a circle [40]. In this study nervous signs were reported in 3 cases in form of ataxia and hind limb paresis, similar results were previously reported [17, 28, 41]. This can be attributed to edematous deposition of hyaluronic acid, resulting in compression of nerve fibers. In canine hypothyroid polyneuropathy, both the cranial nerves and the spinal cord may be compromised. The animal in this study displayed ataxia and hind limbs paresis due to spinal cord alterations [40].

Approximately 45% of hypothyroid dogs had gastrointestinal signs, especially constipation and diarrhea [4] but in our study, two hypothyroid dogs were admitted to the clinic with history of chronic regurgitation of food shortly after food intake with decreased body weight. Radiographic examinations showed a dilatation in thoracic part of esophagus, based on radiographic and thyroid profile findings the dogs were diagnosed for hypothyroidism associated with megaesophagous [42, 43].

This condition may be related to general metabolic disturbances resulting from decreased thyroid function, or it may be a sign of a neuropathy or myopathy [43]. These cases were diagnosed as a secondary condition to hypothyroidism based on clinical improvement after levothyroxine administration [42].

In this study, four hypothyroid dogs showed different gynecological defects such as anestrum, breeding with no conception and galactorrhea, as previously reported [28, 10]. Thyroid hormones deficiency, by negative feedback mechanism induces an increase in TRH secretion, which in turn stimulates prolactin secretion. Increasing level of prolactin hormone, on the other hand, by negative feedback mechanism, induces reduction in estrogen secretion. Infertility problems were resulted from a decrease in estrogen level, which lowers stimulation of FSH and LH and inhibits follicle growth [44].

Treatment with levothyroxine sodium, which is a synthetic form of the thyroid hormone (T4), plays a crucial role in maintaining normal thyroid hormone levels in the body [28].

After administration of levothyroxine for one month, TT4 and FT4 levels were increased while TSH level was decreased compared to hypothyroid group. Clinical improvement was most observed after 6–8 months from the levothyroxine administration [5, 9, 45].

In the treatment of hypothyroidism in dogs, the dosage of thyroid hormone replacement can vary depending on the individual animal. Lower doses may be sufficient for some dogs, as they might not require as much hormone supplementation to achieve normal thyroid function. Additionally, some veterinary experts recommend once-daily dosing, particularly if the dog is stable and the clinical signs are well-controlled. However, twice-daily dosing is often preferred, as it helps to maintain more stable levels of thyroid hormone in the blood. This approach results in less fluctuation in T4 levels, leading to more consistent hormone concentrations throughout the day and avoiding high peak levels that could cause side effects [14].

## Conclusion

Incidence of canine hypothyroidism was reported in 14% of affected dogs that were included in this study. Moreover, Results showed that the highest incidence of hypothyroidism was reported in Golden retriever and middle-aged dogs of different breeds. Males revealed higher incidence than females. When the hypothyroid cases advance, other signs appear including skin abnormalities such as generalized alopecia, myxedema, pyoderma in addition to neuromuscular, cardiovascular, respiratory and gastrointestinal disorders.

This study recommended monitoring of TT4 in dogs with dermatological abnormalities or obesity as a routine test. In addition, further investigation for long term complications of hypothyroidism in larger number of hypothyroid dogs is required.

## Data Availability

All data generated in the study are provided in this manuscript.

## Abbreviations

TT4: Total Tetra-iodothyronine
FT4: Free Tetra-iodothyronine
T.S.H: Thyroid stimulating hormone
LT4: Levothyroxine
E/A ratio: is a ratio of early to late ventricular filling velocities
